# A workflow for mathematical modeling of subcellular metabolic pathways in leaf metabolism of *Arabidopsis thaliana*

**DOI:** 10.3389/fpls.2013.00541

**Published:** 2013-12-24

**Authors:** Thomas Nägele, Wolfram Weckwerth

**Affiliations:** Department of Ecogenomics and Systems Biology, University of ViennaVienna, Austria

**Keywords:** plant systems biology, genome-scale models, metabolomics, inverse calculation, mathematical modeling, subcellular compartmentation

## Abstract

During the last decade genome sequencing has experienced a rapid technological development resulting in numerous sequencing projects and applications in life science. In plant molecular biology, the availability of sequence data on whole genomes has enabled the reconstruction of metabolic networks. Enzymatic reactions are predicted by the sequence information. Pathways arise due to the participation of chemical compounds as substrates and products in these reactions. Although several of these comprehensive networks have been reconstructed for the genetic model plant *Arabidopsis thaliana*, the integration of experimental data is still challenging. Particularly the analysis of subcellular organization of plant cells limits the understanding of regulatory instances in these metabolic networks *in vivo*. In this study, we develop an approach for the functional integration of experimental high-throughput data into such large-scale networks. We present a subcellular metabolic network model comprising 524 metabolic intermediates and 548 metabolic interactions derived from a total of 2769 reactions. We demonstrate how to link the metabolite covariance matrix of different *Arabidopsis thaliana* accessions with the subcellular metabolic network model for the inverse calculation of the biochemical Jacobian, finally resulting in the calculation of a matrix which satisfies a Lyaponov equation. In this way, different strategies of metabolite compartmentation and involved reactions were identified in the accessions when exposed to low temperature.

## Introduction

The rapidly increasing knowledge about whole plant genome sequences represents a corner stone in the understanding of plant metabolism. Next-generation sequencing (NGS) technologies have been developed allowing for the fast and cheap production of huge sets of genome data sequences (Metzker, 2010). By functional genome annotation, the information about coded proteins is derived. Based on these postulated gene functions, enzymatic reactions can be predicted, e.g., representing metabolite interconversions or transport processes. Genome-scale metabolic reconstructions (GEMs) can be described as advanced functional annotations (De Oliveira Dal'molin and Nielsen, 2013). Here, the functional gene annotations are combined with a network topology which is derived by connecting the metabolic educts and products of the predicted reactions. The stoichiometric matrix N characterizes each metabolic interconversion and transition in a metabolic network and is typically organized in such way that rows of the matrix represent the metabolites while reactions, i.e., connections, represent the columns of the matrix. The stoichiometric coefficients of each reaction are then given in the matrix cells. GEMs have been published for multiple organisms (an overview is given in Collakova et al., 2012) and comprehensive protocols for all stages of the reconstruction process are available (Thiele and Palsson, 2010). Due to the huge metabolic coverage of GEMs, which, in principle, comprises all metabolic interactions known so far from genome annotation, strategies for genome-scale experiments are needed for efficient validation of model outputs. In this context, flux measurements and high-throughput measurements of the transcriptome, proteome and metabolome play a crucial role. Hence, technologies like transcriptomics, proteomics and metabolomics are central to systems biology approaches aiming at the system wide understanding of biological networks (Weckwerth, 2011a,b; Blazier and Papin, 2012; Nägele and Weckwerth, 2012). Recently, we connected data from metabolomics experiments to a simplified metabolic network structure of leaf primary metabolism in *Arabidopsis thaliana* to characterize metabolic shifts during cold exposure (Doerfler et al., 2013). This allowed us to differentiate short and long term metabolic response to low temperature and to identify key points of regulation such as the interface of primary and secondary metabolism mediated by the shikimic acid pathway. This model did not include any subcellular compartmentation of metabolism. Plant cells, however, show a high degree of compartmentation resulting in a complex biochemical network comprising metabolite interconversions as well as intracellular transport processes (Lunn, 2007). Therefore it is not surprising that physiological responses to a changing environment could be related to subcellular metabolic reprogramming (Knaupp et al., 2011; Schulze et al., 2012; Nägele and Heyer, 2013). To enhance the comprehensive biochemical and physiological output of studies analyzing plant-environment interactions a platform would be desirable focusing the linkage of experimental data with the underlying subcellular metabolic network structure.

Here, we present a workflow aiming at the development of such a platform. Based on a recently published metabolic network reconstruction accounting for subcellular organization of leaf metabolism in *Arabidosis thaliana* (Mintz-Oron et al., 2012), we derived a metabolic model structure including all metabolic intermediates which are accessible by experimental high-throughput measurements. The model comprises all subcellular compartments of leaf cells which can be robustly analyzed by a non-aqueous fractionation technique. For efficient data integration we present a strategy of mathematical modeling which we prove to be successful in providing a comprehensive overview of metabolic changes induced by a perturbed plant-environment interaction.

## Material and methods

### Adaptation of a genome-scale metabolic reconstruction model to a set of experimentally accessible data

The model adaptation was performed starting with the original metabolic reconstruction model for (juvenile) leaf metabolism, which was derived by Mintz-Oron and co-workers (Mintz-Oron et al., 2012). This reconstruction model comprises 7 compartments which are the cytoplasm, endoplasmic reticulum, golgi apparatus, mitochondrion, peroxisome, plastid and vacuole with a total of 2463 metabolic intermediates and 2769 reactions (Mintz-Oron et al., 2012). In a first step, we reduced the compartments in the model to the cytoplasm, plastid and vacuole which can experimentally be analyzed from the same sample using the non-aqueous fractionation (NAF) method (Nägele and Heyer, 2013). In a second step, we reduced the metabolic intermediates of these compartments to a set of metabolites which are experimentally accessible by a gas chromatography coupled to mass spectrometry (GC-MS) analysis. This reduction step was performed by comparison of metabolites in the model to metabolites in the Golm Metabolome Database for GC-MS based metabolite profiling (Hummel et al., 2007). Additionally, the model contains the plastidial starch pool as well as CO_2_. In the following step, all intermediates in the reduced model were (re)connected manually according to the reactions described in the original reconstruction model and no further reactions were added. Hence, the reduced model describes a subset of the metabolic connections in the original model with a lower degree of detail. A step of this reduction procedure is exemplarily shown in Figure S1. Finally, our reduced model contained 3 compartments, 524 metabolic intermediates and 548 metabolic interactions. The reduced model of leaf metabolism is provided in the supplements in Systems Biology Markup Language (SBML; File ‘Model_S3.xml’). Model reduction, connection and graphical evaluation was performed using the open source software COPASI (Version 4.8; http://www.copasi.org) (Hoops et al., 2006) and CellDesigner™ (Version 4.3; http://www.celldesigner.org/) (Funahashi et al., 2003).

### Data integration and jacobian matrix calculation

A metabolic interaction matrix was derived from the reduced model describing all metabolic interactions in the model. Hence, the metabolic interaction matrix represents a simplified version of the stoichiometric matrix of the original metabolic reconstruction model. This metabolic interaction matrix was applied for inverse calculation of the Jacobian matrix of the metabolic model. The calculation procedure was based on an algorithm, which is implemented in the metabolomics toolbox COVAIN (Sun and Weckwerth, 2012), solving (Eq. 1) by applying a total least square optimization procedure. To test the reliability of our calculations we applied the inverse calculation on a data set on subcellular carbohydrate distribution from non-cold exposed and 7 days cold exposed Arabidopsis leaf samples which were published recently (Nägele and Heyer, 2013). This experimental data set contained 5 biological replicates for each metabolite concentration (Nägele and Heyer, 2013). The reduced model structure, i.e., the metabolic interaction matrix, was adapted to the metabolite pools which had been experimentally analyzed by Nägele and Heyer. The covariance matrix C was built from the experimental data on subcellular carbohydrate concentration and linked to the underlying biochemical system by the following equation (Steuer et al., 2003; Sun and Weckwerth, 2012):

(1) $$JC+CJ^{T}=-2D$$

Here, J represents the Jacobian matrix and D is the fluctuation matrix. All diagonal entries of D were randomly drawn from a standard normal distribution. In general, the Jacobian matrix characterizes the local dynamics at a steady state condition. In context of a metabolic network, entries of the Jacobian J represent the elasticities of reaction rates to any change of the metabolite concentrations which are characterized by equation 2:

(2) $$J=N\frac{\partial r}{\partial M}$$

N is the stoichiometric matrix, r represents the rates for each reaction and M represents metabolite concentrations. In our approach, we replaced the stoichiometric matrix N by the reduced metabolic interaction matrix. This results in a Jacobian matrix referring to the underlying stoichiometric simplification. Although N was changed, the term Jacobian matrix can still be used as it directly refers to the first partial derivative of (now simplified) metabolic functions to changes in metabolite levels. This corresponds to the general definition of the Jacobian matrix in context of the mean value theorem of vector functions, which is provided elsewhere (Strehmel et al., 2012). The Jacobian matrices J_*a*_ and J_*b*_, which describe two different metabolic states, were calculated 10^5^ times each. Medians of calculated Jacobians were normalized to the square of the interquartile distance in order to increase the median-to-noise ratio of the inverse calculations (Eq. 1). To compare two different metabolic states, we determined the absolute values of the differential Jacobian, dJ_ij, abs_, defining the relative change of the two normalized Jacobians J_a,norm_ and J_b,norm_ which are associated with different treatments or genotypes:

(3) $$dJ_{ij,\;abs}=\left|log_{2}\left|\frac{J_{\text{a},\text{ norm},\text{ ij}}}{J_{\text{b},\text{ norm},\text{ ij}}}\right|\right|$$

All calculations of Jacobian matrices as well as statistical analyses (*t*-tests) were performed using the numerical software environment Matlab® (V7.12.0 R2011a).

## Results

### A subcellular metabolic network model comprising experimentally accessible intermediates from high-throughput analysis of one sample

The reduction process of the metabolic reconstruction network of leaf metabolism (Mintz-Oron et al., 2012) resulted in a metabolic network model comprising the compartments cytoplasm, plastid and vacuole with a total of 524 metabolic intermediates and 548 metabolic interactions. In addition to metabolite pools which are accessible by a GC-MS experiment, the model also comprises the plastidial starch pool and CO_2_. These intermediates are experimentally accessible by, for example, GC-MS analysis of starch hydrolysate, photometric assays (starch) and infrared gas analysis (CO_2_) as previously described (Wienkoop et al., 2010; Nägele and Heyer, 2013; Valledor et al., 2013).

While the metabolic network reconstruction of subcellular leaf metabolism resulted in a stoichiometric matrix derived from genome sequence information (Mintz-Oron et al., 2012), this stoichiometric matrix was changed during the reduction process by deleting and reconnecting components. Hence, the resulting matrix of the reduced model differs a lot from the original stoichiometric information and therefore it is termed as the metabolic interaction matrix. It describes all experimentally accessible metabolic interactions by superpathways. These superpathways implicitly describe all metabolic steps which are involved in a metabolic interaction. If all metabolic intermediates of a reaction or pathway are included in both the original and reduced model then the entries of the metabolic interaction matrix equal the entries of the stoichiometric matrix.

### Application of the reduced metabolic interaction matrix to analyse regulation of carbohydrate compartmentation

**Figure 1:**
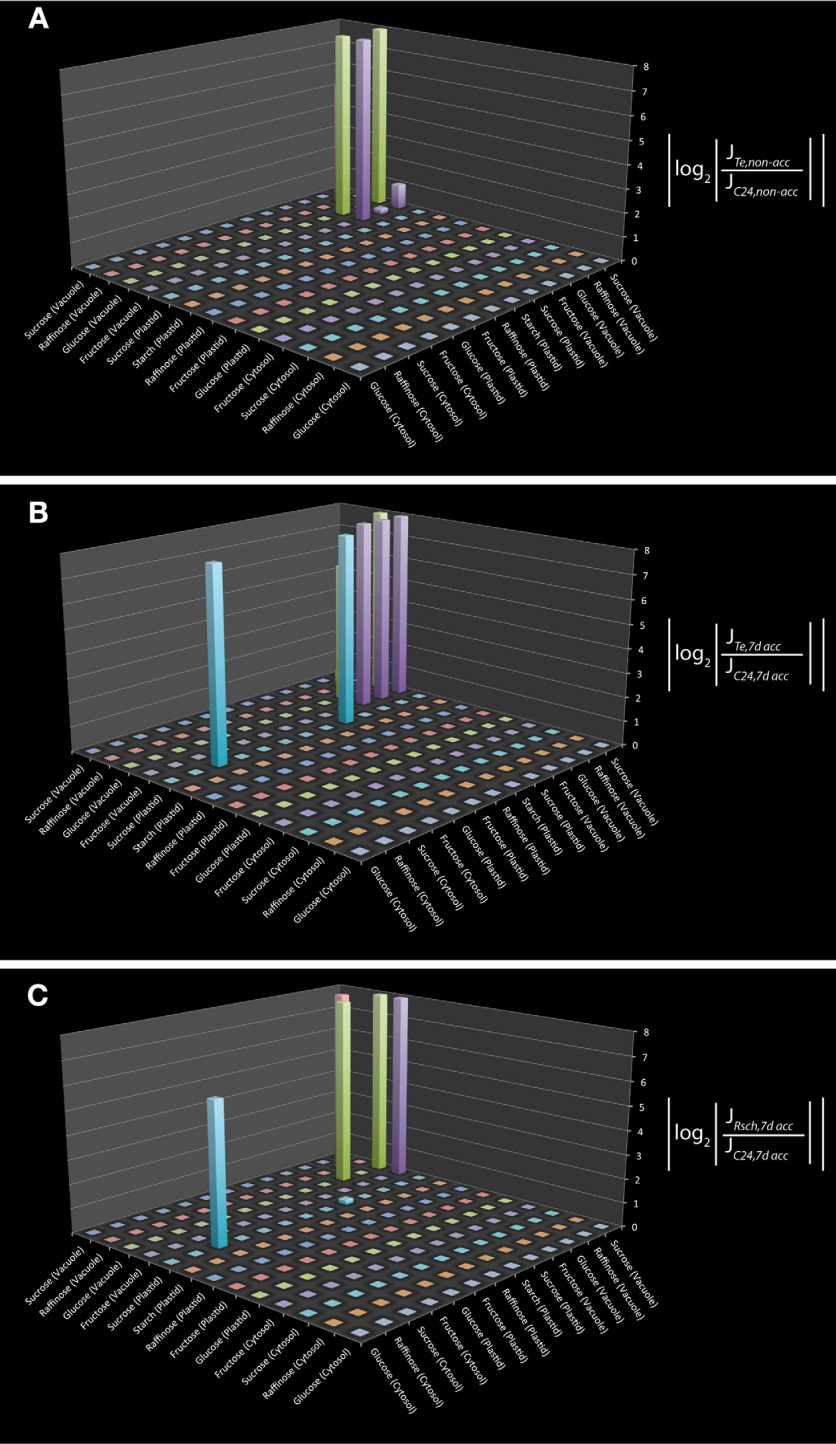
**Differential Jacobian matrices derived from inverse calculations on subcellular carbohydrate concentrations.** Differential Jacobians were built for Te and C24 before **(A)**, and after **(B)** 7 days of cold exposure. For Rsch and C24, the differential Jacobian was built for 7 day cold exposed samples **(C)**. Experimental data were taken from a previous study (Nägele and Heyer, 2013). Metabolic interaction sites are indicated on the horizontal x- and y-axis by the metabolites which participate in the reaction that is characterized by the entry of the Jacobian matrix. For example, in **(B)** the interaction of plastidial and cytosolic sucrose is significantly different between Te and C24 (non-diagonal blue bar). This can also be observed in **(C)** but not in **(A)**.

To test the applicability of the reduced model structure to analyse subcellular metabolic interaction, we used the underlying metabolic interaction matrix for inverse calculation of Jacobian matrices to a recently published data set on subcellular carbohydrate compartmentation (Nägele and Heyer, 2013). We derived a specific metabolic interaction matrix which contained only those pools which were measured by Nägele and Heyer by further reduction of the metabolic network structure. Finally, the model described the pools of sucrose, raffinose, glucose, fructose and starch in the cyotsol, plastid and vacuole as well as their metabolic interactions. In addition to these measured pools we also included all direct metabolic interactions which were not experimentally analyzed, i.e., all metabolites which were connected to the measured pools by one further metabolic interaction. Including those interactions may be helpful for estimating the impact of modeling results with respect to the metabolite interconversions which are directly connected to the set of metabolites which were experimentally analyzed. Yet, this model extension does not affect the modeling results but is rather suggested to support the interpretation. The resulting metabolic interaction matrix describing all reactions and transports of the measured metabolites was used for the inverse calculation of a Jacobian matrix. Calculations were performed exemplarily on experimental data sets for three natural accessions of *Arabidopsis thaliana*, C24, Rschew (Rsch) and Tenela (Te) before (non-acc) and after a 7 day exposure to 4°C (7d acc) (Nägele and Heyer, 2013). Results of inverse calculations indicated a main difference between C24 and Te to exist in vacuolar hexose interactions as well as in the cytosolic and plastidial sucrose interaction (Figures 1 A,B; *p* < 0.05). Comparison of calculations for 7d acc samples of C24 and Rsch revealed a similar and still significant interaction pattern, but to a lower extent (Figure 1C; *p* < 0.05). For non-acc samples, the comparison between C24 and Rsch revealed no further signficant differences than the comparison of C24 and Te (data not shown). To exclude the possiblity that these differences in metabolic interactions represent artifacts from the model reduction process itself, the reactions in the reduced model structure were compared to the original metabolic reconstruction work (Table S2, yellow marked lines). In the reduced model, these interactions still correspond to the original model and were not a consequence of inserted reactions. Hence, they are directly linked to the enzymatic reactions described in the original metabolic network reconstruction.

## Discussion

The development of genome-scale metabolic network models has become a central approach to approximate the topology of metabolic networks *in vivo*. While such networks have successfully been applied in several studies to analyse network properties (Poolman et al., 2009) or to derive strategies of metabolic engineering (Feist and Palsson, 2008), experimental validation of the model output is still limiting. Frequently, this results in a descriptive and qualitative network model which is useful for many purposes but lacks of a predictive output. To overcome this limitation, merging kinetic or stoichiometric modeling and genome-scale metabolic network models represents a promising approach. For kinetic modeling, it is necessary to reduce the metabolic system of interest to an extent which allows the absolute quantification of all participating metabolic intermediates and enzyme kinetic parameters. Although there exist platforms for measuring enzyme activities in an automatized and high-throughput manner (Gibon et al., 2004), it is still highly laborious to robustly resolve systems dynamics which allow for an unambigous output of computational model simulation (Schaber et al., 2009). Yet, despite these limitations, the output of such enzyme kinetic models has been shown to significantly promote the understanding of complex biological issues (Nägele et al., 2012; Rohwer, 2012). Stoichiometric modeling, in contrast, relies rather on the stoichiometry of a network than on detailed kinetic information about enzymatic interconversions. Although the metabolic coverage of stoichiometric models is, in general, much larger than in enzyme kinetic models, systems dynamics can, very often, only be approximated by linearization of metabolite functions, i.e., reaction rates, at a certain metabolic steady state. This results in the Jacobian matrix which characterizes the dynamic capabilities at these steady states (Steuer, 2007) and represents the elasticities of reaction rates to any change of the metabolite concentrations. Hence, to determine the entries of the Jacobian matrix explicit knowledge about enzyme kinetics is needed, which can, again, hardly be determined for a metabolic network comprising several hundred or even thousands of metabolic interactions. Instead, the inverse approximation of the Jacobian entries from metabolomics (co-)variance can be applied and directly links experimental data with stoichiometric information on a metabolic network (Weckwerth, 2011b; Sun and Weckwerth, 2012; Doerfler et al., 2013).

Our reduced metabolic model of subcellular primary leaf metabolism in *Arabidopsis thaliana* intends to provide a platform aiming at the integration of as many as possible experimental data to metabolic network information. Although the experimental coverage of metabolic intermediates may significantly deviate from the metabolic coverage of the model, the model can be adjusted to the available experimental data set. We have exemplarily performed such an adjustment to an experimental data set on the central carbohydrate metabolism which was published previously (Nägele and Heyer, 2013). Results of the inverse calculation of the Jacobian matrix indicated a main difference between cold sensitive and tolerant accessions of Arabidopsis to exist in the ability to transport sucrose across the chloroplast envelope. This agrees with the predictions made previously, applying a different approach of metabolic modeling (Nägele and Heyer, 2013). In addition, inverse calculations also pointed to a differential regulation of vacuolar hexose interaction, i.e., the interconversion and transport of vacuolar glucose and fructose, which were also found to be signficantly affected due to cold exposure in a previous study (Wormit et al., 2006). However, in this context, we want to emphasize that because of the reduction step it is not possible to give an interpretation of the differential Jacobian in terms of absolute fluxes or rates of metabolic reactions. Based on the covariance matrix of relative metabolite levels, entries of the differential Jacobian matrix only report on a qualitative perturbation of a certain metabolic interaction, i.e., changes in the Jacobian entries between two conditions or genotypes. This directly corresponds to the so-called community matrix *A* which was introduced to describe species-species interactions in an ecosystem (May, 1974). Its elements *a_ij_* describe the net effect of species *j* on species *i* near equilibrium. Likewise, entries of the differential Jacobian *dJ_ij_* describe changes in the interaction between metabolite *j* with respect to changes in metabolite *i* due to a perturbation of the metabolic system.

**Figure 2:**
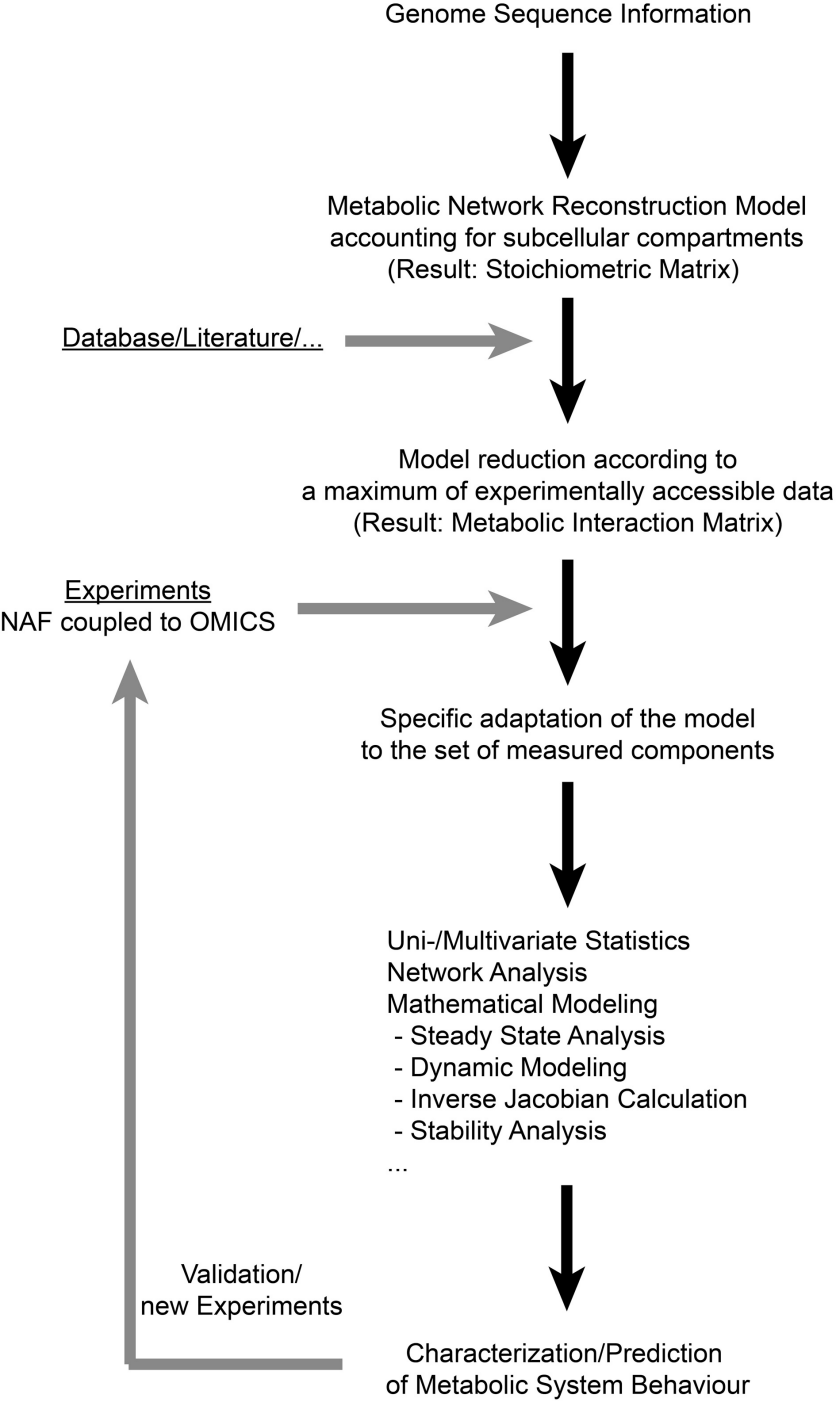
**A workflow for deriving subcellular metabolic network structures according to experimental data sets.** In the present study, the first step of deriving a genome-scale metabolic network reconstruction model was not performed, but a published reconstruction work was applied instead (Mintz-Oron et al., 2012). NAF: Non-Aqueous Fractionation.

Previous studies have shown that NAF coupled to high-throughput analysis enables the comprehensive characterization of a metabolic homeostasis (Klie et al., 2011; Krueger et al., 2011). Such comprehensive experimental approaches result in huge and multidimensional data sets, comprising information about numerous variables, for example subcellular metabolite levels. Besides experimental high-throughput analysis, systems biology ultimately attempts to exploit the large calculating capacities of computers to efficiently cope with the large data sets covering complex interactions. Computer based handling of complex metabolic networks requires their formal representation by mathematical models. The integration of experimental data then allows for the *in silico* simulation of specific responses, and predictions can be validated in further experiments. Hence, in an iterative process of model development, model simulation and experimental validation, systems biology is capable of advancing the understanding of complex networks significantly. Based on these requirements for data integration and the results of the present study, we suggest a workflow for the functional integration of experimental high-throughput data to the metabolic network structure of the subcellular leaf metabolism of *Arabidopsis thaliana* (Figure 2). Applying the genome sequence information and a deduced metabolic network reconstruction model, a metabolic interaction matrix can be constructed allowing for the functional integration of experimental data on subcellular metabolite levels. As experimental data sets may vary in the components they comprise, the model structure can be specifically adapted to the available experimental data set which makes this approach universally applicable to various (bio-) analytical methods. This enables the application of methods from systems theory and applied mathematics (Nägele and Weckwerth, 2013) which are essential for the evaluation of complex biological systems, such as the compartmentalized leaf metabolism of Arabidopsis. Finally, hypotheses about regulation of subcellular metabolic interactions can be tested in a new experimental design which completes the iterative cycle of model predicitons and experiments, being a general characteristic of systems biology approaches.

## Author contributions

Thomas Nägele performed model programming, calculation, modeling and wrote the manuscript. Wolfram Weckwerth wrote the manuscript. Thomas Nägele and Wolfram Weckwerth performed the design of the study.

## Conflict of interest statement

The authors declare that the research was conducted in the absence of any commercial or financial relationships that could be construed as a potential conflict of interest.

## Supplementary material

Figure S1 **Schematic overview of the model reduction process.** In contrast to metabolite **(B)**, metabolites **(A)** and **(C)** are accessible by a GC-MS measurement and are kept in the reduced model structure.

Table S2 **Reactions included in the reduced model (column B) are compared to reactions of the original reconstruction network (column E).** Reactions which have been identified to be affected differentially during cold exposure of sensitive and tolerant Arabidopsis accessions are marked in yellow (referring to Figure 1).

Model S3 **Reduced model structure in format of Systems Biology Markup Language (xml-file)**.
