# Supplementary material for: A workflow for mathematical modeling of subcellular metabolic pathways in leaf metabolism of Arabidopsis thaliana
Source: Front Plant Sci. 2013 Dec 24;4:541. doi: 10.3389/fpls.2013.00541 (PMC3872044; doi:10.3389/fpls.2013.00541)
Supplement: Figure S1 — Schematic overview of the model reduction process. In contrast to metabolite (B), metabolites (A) and (C) are accessible by a GC-MS measurement and are kept in the reduced model structure. [file DataSheet1.PDF]

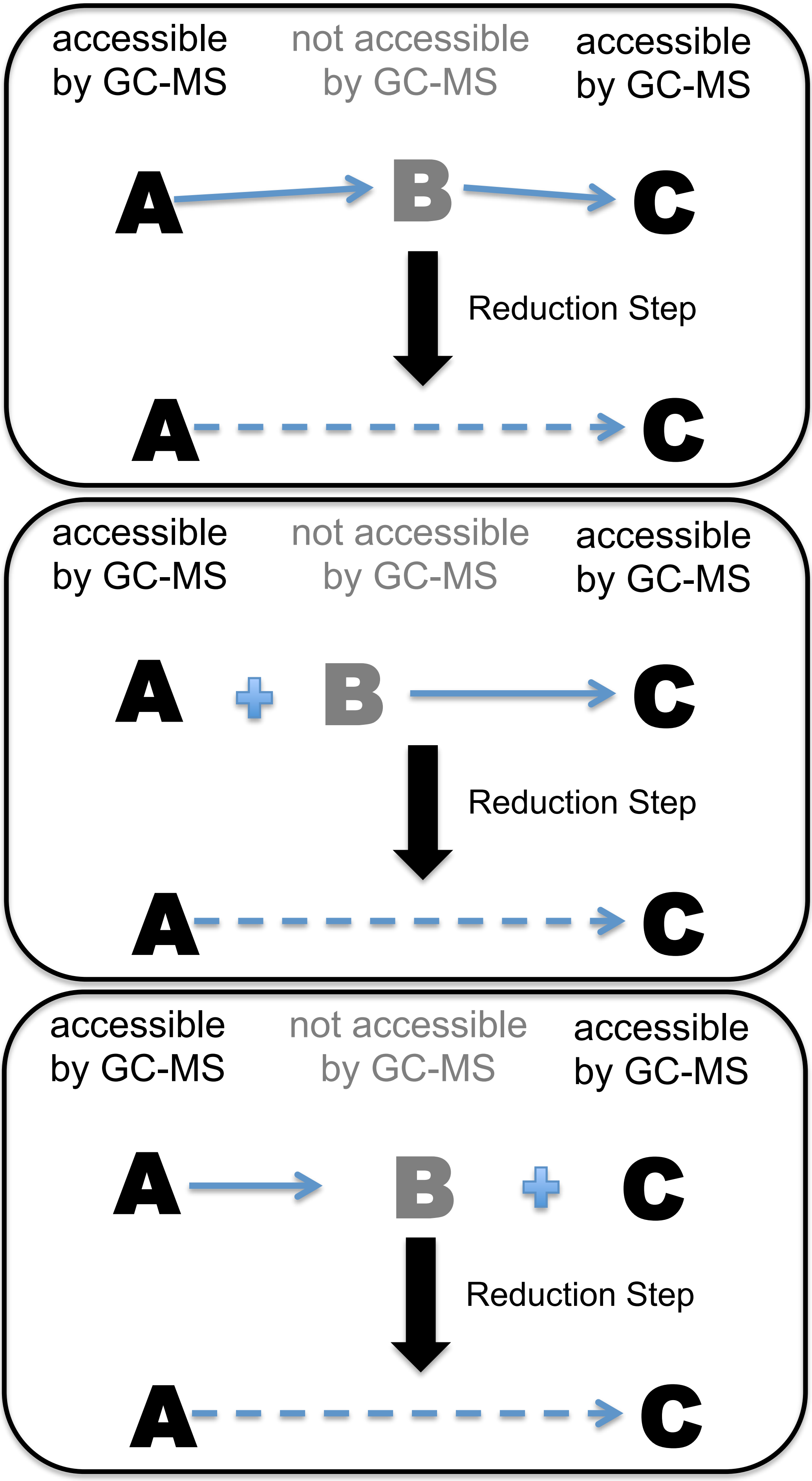
accessible by GC-MS
not accessible by GC-MS
accessible by GC-MS
A
B
C
Reduction Step
A
C
accessible by GC-MS
not accessible by GC-MS
accessible by GC-MS
A
B
C
Reduction Step
A
C
accessible by GC-MS
not accessible by GC-MS
accessible by GC-MS
A
B
C
Reduction Step
A
C
